# Multicentre phase II study of nivolumab in Japanese patients with advanced or recurrent non-squamous non-small cell lung cancer

**DOI:** 10.1136/esmoopen-2016-000108

**Published:** 2017-03-07

**Authors:** Makoto Nishio, Toyoaki Hida, Shinji Atagi, Hiroshi Sakai, Kazuhiko Nakagawa, Toshiaki Takahashi, Naoyuki Nogami, Hideo Saka, Mitsuhiro Takenoyama, Makoto Maemondo, Yuichiro Ohe, Hiroshi Nokihara, Tomonori Hirashima, Hiroshi Tanaka, Shiro Fujita, Koji Takeda, Koichi Goto, Miyako Satouchi, Hiroshi Isobe, Koichi Minato, Naoki Sumiyoshi, Tomohide Tamura

**Affiliations:** 1 Department of Thoracic Medical Oncology, The Cancer Institute Hospital, Japanese Foundation for Cancer Research, Tokyo, Japan; 2 Department of Thoracic Oncology, Aichi Cancer Center Hospital, Aichi, Japan; 3 Department of Internal Medicine, National Hospital Organization Kinki-Chuo Chest Medical Center, Osaka, Japan; 4 Department of Thoracic Oncology, Saitama Cancer Center, Saitama, Japan; 5 Department of Medical Oncology, Kindai University Faculty of Medicine, Osaka, Japan; 6 Division of Thoracic Oncology, Shizuoka Cancer Center, Shizuoka, Japan; 7 Department of Thoracic Oncology and Medicine, National Hospital Organization Shikoku Cancer Center, Ehime, Japan; 8 Department of Medical Oncology, National Hospital Organization Nagoya Medical Center, Aichi, Japan; 9 Department of Thoracic Oncology, National Hospital Organization Kyushu Cancer Center, Fukuoka, Japan; 10 Department of Respiratory Medicine, Miyagi Cancer Center, Miyagi, Japan; 11 Division of Thoracic Oncology, National Cancer Center Hospital, Tokyo, Japan; 12 Department of Thoracic Malignancy, Osaka Prefectural Medical Center for Respiratory and Allergic Diseases, Osaka, Japan; 13 Department of Internal Medicine, Niigata Cancer Center Hospital, Niigata, Japan; 14 Division of Integrated Oncology, Institute of Biomedical Research and Innovation hospital, Hyogo, Japan; 15 Department of Medical Oncology, Osaka City General Hospital, Osaka, Japan; 16 Department of Thoracic Oncology, National Cancer Center Hospital East, Chiba, Japan; 17 Department of Thoracic Oncology, Hyogo Cancer Center, Hyogo, Japan; 18 Department of Medical Oncology, KKR Sapporo Medical Center, Hokkaido, Japan; 19 Division of Respiratory Medicine, Gunma Prefectural Cancer Center, Gunma, Japan; 20 Oncology Clinical Development Planning 1, Ono Pharmaceutical Co. Ltd., Osaka, Japan; 21 Thoracic Center, St. Luke's International Hospital, Tokyo, Japan

**Keywords:** nivolumab, non-small cell lung cancer, programmed cell death-1, non-squamous histology, PD-L1

## Abstract

**Objective:**

Nivolumab is a fully human IgG4 programmed cell death 1 immune checkpoint inhibitor monoclonal antibody approved for the treatment of non-small cell lung cancer (NSCLC). The aim of this study was to evaluate the safety and efficacy of nivolumab in Japanese patients with advanced or recurrent non-squamous NSCLC.

**Methods:**

In this multicentre phase II study, patients with advanced or recurrent non-squamous NSCLC, which had progressed after platinum-containing chemotherapy, were treated with nivolumab 3 mg/kg, intravenously every 2 weeks until progressive disease or unacceptable toxicity was observed. The primary end point was independent radiology review committee (IRC) assessed overall response rate (ORR) and the secondary endpoints included ORR (investigator assessed), progression-free survival (PFS), overall survival (OS), duration of response, time to response, best overall response, and safety.

**Results:**

76 patients were enrolled across 19 sites in Japan. The ORR (IRC assessed) was 22.4% (95% CI 14.5% to 32.9%). The median PFS and OS were 2.8 months (95% CI 1.4 to 3.4) and 17.1 months (95% CI 13.3 to 23.0), respectively. The OS rate at 1 year was 68.0% (95% CI 56.2% to 77.3%). Current/former smokers were more responsive to treatment than non-smokers (ORR 29.1% vs 4.8%). Patients with epidermal growth factor receptor (EGFR) mutation wild type/unknown showed higher ORR compared with EGFR mutation-positive patients (ORR 28.6% vs 5.0%) and programmed cell death ligand-1 (PD-L1) expression was likely associated with higher ORR, longer PFS and OS. Treatment-related adverse events of grade 3 or higher were reported in 17 patients; these events resolved or were resolving with appropriate treatment including steroid therapy or discontinuation of nivolumab.

**Conclusions:**

Nivolumab was well tolerated and showed clinical efficacy in Japanese patients with non-squamous NSCLC progressed after platinum-containing chemotherapy, especially in those with a history of smoking, wild type/unknown EGFR mutation status or positive PD-L1 expression.

**Trial registration number:**

JapicCTI-132073.

**Key questions**

**What is already known about this subject?**

- Nivolumab is a fully human IgG4 programmed death receptor-1 blocking antibody.
- It is currently approved for the treatment of non-small cell lung cancer (NSCLC).

**What does this study add?**

- Nivolumab is associated with clinical efficacy and manageable tolerability in Japanese patients with advanced/recurrent non-squamous NSCLC.
- Patients with programmed cell death ligand-1-positive, current or former smokers, and patients with epidermal growth factor receptor (EGFR) mutation wild type/unknown can experience greater benefit with nivolumab.

**How might this impact on clinical practice?**

- Nivolumab may be effectively used in the treatment of Japanese patients with advanced/recurrent non-squamous NSCLC and further studies with a larger patient population are warranted.

## Introduction

Lung cancer is a leading cause of cancer-related deaths worldwide. In Japan, 113 237 cases of malignant neoplasm of the lungs are reported annually, with death reported in 73 396 of these cases.^1^ ^2^ Non-small cell lung cancer (NSCLC) accounts for 85–90% of the reported lung cancer cases and is further classified based on histology as squamous or non-squamous NSCLC.^3^ Treatment of stage IIIB/IV or recurrent NSCLC progressed after platinum-containing chemotherapy remains a challenge as therapeutic efficacy of currently available treatment regimens is limited.^4–6^

Programmed cell death 1 (PD-1) is a receptor expressed on the surface of activated T cells;^7^ it binds to its ligands programmed cell death ligand-1 (PD-L1) and PD-L2, causing downregulation of the activated T cells.^8^ Expression of PD-L1 in NSCLC generates an immunosuppressive tumour microenvironment and promotes tumour immune escape, thus leading to poor prognosis of the disease.^9^ Nivolumab is a fully human IgG4 anti-PD-1 blocking monoclonal antibody approved for the treatment of NSCLC. The randomised phase III CheckMate-057 study conducted globally except in Japan, Korea and Taiwan compared nivolumab with docetaxel in patients with advanced non-squamous NSCLC with disease progression during or after platinum-containing doublet chemotherapy and reported superior efficacy and tolerability of nivolumab over docetaxel.^10^ The present study evaluated the safety and efficacy of nivolumab in Japanese patients with advanced non-squamous NSCLC, which progressed after platinum-containing chemotherapy.

## Patients and methods

### Study design

This multicentre, open-label, phase II study (see online supplementary figure S1A) conducted in Japan enrolled adults (aged ≥20 years), with Eastern Cooperative Oncology Group (ECOG) performance status of 0–1, histologically or cytologically confirmed stage IIIB/IV non-squamous NSCLC (according to Union for International Cancer Control-TNM Classifications, Seventh Edition^11^) or recurrent Response Evaluation Criteria in Solid Tumor (RECIST) after surgical resection. Eligible patients had (1) ≥1 measurable lesion by RECIST criteria (V.1.1); (2) a history of prior treatment with a platinum-containing chemotherapy regimen for patients with epidermal growth factor receptor (EGFR) mutation wild type/unknown and an anaplastic lymphoma kinase (ALK) translocation negative/unknown; or two regimens of platinum-containing chemotherapy and any EGFR tyrosine kinase inhibitor for EGFR mutation-positive patients or two regimens of platinum-containing chemotherapy and any ALK inhibitor for ALK translocation-positive patients and percutaneous oxygen saturation (SpO_2_) ≥94%. Treatment could continue beyond initial disease progression if the investigator assessed that the patient was having clinical benefit and did not have an unacceptable level of side effects from the study drug.

10.1136/esmoopen-2016-000108.supp1 supplementary figures and tables

Patients received nivolumab 3 mg/kg administered intravenously every 2 weeks in each 6-week cycle until radiographically confirmed disease progression, unacceptable toxicity, withdrawal, or death. The study was approved by the Institutional Review Board of each study site and was conducted in accordance with the ethical principles of the Declaration of Helsinki. Written informed consent was obtained from all patients prior to the study. The data cut-off date was 17 December 2015 (Clinical trial registration: JapicCTI-132073).

### Efficacy assessments

Tumour response was assessed during the treatment and follow-up periods at predefined time points according to the RECIST guidelines (V.1.1). The primary end point was the independent radiology review committee (IRC) assessed confirmed overall response rate (ORR). Secondary end points included confirmed ORR (investigator assessed), progression-free survival (PFS), overall survival (OS), duration of response (DOR), time to response, best overall response (BOR) and change in tumour size.

### Safety assessments

Adverse events (AEs) reported during the study were rated using the National Cancer Institute's Common Terminology Criteria for Adverse Events, V.4.0. Select AEs (those with a potential immunological cause) were grouped according to prespecified categories.

### Subgroup analysis

A prespecified subgroup analysis for ORR and a post-hoc subgroup analysis for PFS and OS were performed to determine the association between these efficacy variables and the patients' age, gender, ECOG performance status, brain metastasis, disease stage, smoking status, and EGFR mutation status.

### PD-L1 analysis

Tumour PD-L1 expression was assessed retrospectively in pretreatment (archival or recent) tumour-biopsy specimens using a validated, automated immunohistochemical assay (Dako North America) that used a rabbit anti-human PD-L1 antibody (clone 28–8, Epitomics). Tumour PD-L1 expression was confirmed when the tumour cell membranes were stained (at any intensity) at predetermined expression levels of ≥1%, ≥5%, and ≥10% in a section that included at least 100 tumour cells that could be evaluated.

### Statistical analysis

The expected response rate for nivolumab was set at 20%. Assuming a threshold response rate of 9%,^6^ 67 patients were needed in order to ensure a power of ≥80% at a one-sided significance level of 0.025 in binominal tests (normal approximation). If a tumour response was achieved in at least 11 of the 67 patients, the null hypothesis was rejected. The study planned to enrol 75 patients to allow for non-evaluable patients.

The baseline characteristics of patients enrolled in the study were summarised using frequency distributions and summary statistics. The primary efficacy and safety analyses were conducted in all patients who received at least one dose of nivolumab. AEs and Grade observed between the date of the first administration of nivolumab and 28 days after the last dose or the start of subsequence anti-cancer therapy after the last dose, whichever comes first, were tabulated.

## Results

The study enrolled 76 patients from 19 sites in Japan between April and October 2013 (table 1, online supplementary figure S1B). The number of patients with EGFR mutation-positive status was 20 (26.3%). After discontinuation of treatment, 53.9% of the patients received subsequent systemic cancer therapy. 21.1% of the patients received subsequent docetaxel (see online supplementary table S7).

**Table 1:** Baseline characteristics and prior treatment received by the study patients

Baseline characteristics	N=76
Age, years	
Median	64.0
Range	39–78
Age category, n (%) (years)	
<65	40 (52.6)
≥65	36 (47.4)
Gender, n (%)	
Males	49 (64.5)
Females	27 (35.5)
ECOG PS, n (%)	
0	28 (36.8)
1	48 (63.2)
Tumour type, n (%)	
Large cell carcinoma	2 (2.6)
Adenocarcinoma	74 (97.4)
Disease stage, n (%)	
IIIB	0 (0.0)
IV	62 (81.6)
Recurrent	14 (18.4)
Brain metastasis, n (%)	
Yes	21 (27.6)
No	55 (72.4)
Prior systemic regimens, n (%)	
1	57 (75.0)
2	19 (25.0)
Smoking status, n (%)	
Never	21 (27.6)
Former	51 (67.1)
Current	4 (5.3)
EGFR mutation status, n (%)	
Positive	20 (26.3)
Wild type or unknown	56 (73.7)
Prior treatment, n (%)	
Platinum-containing chemotherapy	76 (100.0)
Carboplatin	40 (52.6)
Cisplatin	34 (44.7)
Carboplatin+cisplatin	2 (2.6)
EGFR tyrosine kinase inhibitor	20 (26.3)
Gefitinib	12 (15.8)
Erlotinib	7 (9.2)
Afatinib	1 (1.3)

ECOG, Eastern Cooperative Oncology Group; EGFR, epidermal growth factor receptor; PS, performance status.

### Efficacy

The IRC-assessed and investigator-assessed ORRs were 22.4% (95% CI 14.5% to 32.9%) and 25.0% (95% CI 16.6% to 35.8%), respectively (table 2). The IRC assessed disease control rate (complete response (CR)+partial response (PR)+stable disease (SD)) was 47.4%.

**Table 2:** Tumour response and survival in patients with advanced non-squamous NSCLC treated with nivolumab

Best overall response	IRC assessed, n (%)	Investigator assessed, n (%)
CR	2 (2.6)	1 (1.3)
PR	15 (19.7)	18 (23.7)
Stable disease	19 (25.0)	17 (22.4)
Progressive disease	38 (50.0)	40 (52.6)
Not evaluable	1 (1.3)	0 (0)
No measurable lesion	1 (1.3)	0 (0)
ORR (CR+PR), % (95% CI)	17, 22.4% (14.5% to 32.9%)	19, 25.0% (16.6% to 35.8%)
Progression-free survival (IRC assessed)		
Median, months (95% CI)	2.8 (1.4 to 3.4)	
Range, months	0.4–31.4*	
Rate at 1 year, % (95% CI)	24.2 (14.9 to 34.7)	
Overall survival		
Median, months (95% CI)	17.1 (13.3 to 23.0)	
Range, months	0.9–31.9*	
Rate at 1 year, % (95% CI)	68.0 (56.2 to 77.3)	
Time to response		
Responders, n	17	
Median, months (range)	1.4 (1.3–14.8)	
Duration of response		
Median, months (range)	NR (1.6*–29.1*)	

*A censored value.

CR, complete response; IRC, independent radiology review committee; NR, not reached; NSCLC, non-small cell lung cancer; ORR, overall response rate; PR, partial response.

**Figure 1:**
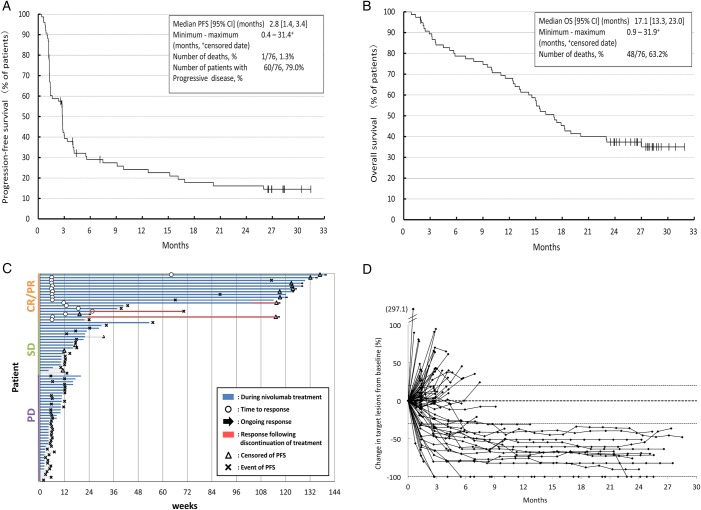
Efficacy of nivolumab in Japanese patients with advanced non-squamous non-small cell lung cancer (A) Kaplan-Meier curve for PFS, (B) Kaplan-Meier curve for OS, (C) duration of response (arrows indicate censored data), and (D) change in tumour size from baseline. CR, complete response; OS, overall survival; PD, progressive disease; PFS, progression-free survival; PR, partial response; SD, stable disease.

The lower limit of the 95% CI of the ORR with nivolumab exceeded the threshold response rate of 9% (null hypothesis) which was based on the ORR (8.8%) for docetaxel.^6^ No substantial difference was observed between the IRC and investigators' assessment of PFS (median PFS (IRC assessment) 2.8 months (95% CI 1.4 to 3.4); (investigator's assessment) 2.8 months (95% CI 1.4 to 3.4); figure 1A). The median OS was 17.1 months (95% CI 13.3 to 23.0; figure 1B), corresponding with an OS rate at 1 year of 68.0% (95% CI 56.2% to 77.3%). Tumour size shrinkage was observed in >50% of patients and the median DOR was not reached (range 1.6–29.1 months; figure 1C, D). The median time to response was 1.4 months (range 1.3–14.8) in the 17 patients with treatment response. The median follow-up for OS was 16.6 months (range 0.9–31.9).

### Safety

Treatment-related AEs were observed in 64 patients (84.2%) during the study (table 3). Treatment-related AEs reported in ≥10% of patients were malaise (n=11, 14.5%), had pyrexia (n=11, 14.5%), rash (n=11, 14.5%), decreased appetite (n=11, 14.5%), fatigue (n=9, 11.8%), nausea (n=9, 11.8%), and pruritus (n=8, 10.5%). Events in which interstitial shadows was confirmed by chest CT were reported in six patients (interstitial lung disease (n=4, 5.3%), lung disorder (n=2, 2.6%)). Treatment-related AEs of ≥grade 3 were observed in 17 patients (22.4%) and included interstitial lung disease, colitis, liver disorder, and pruritus (table 3).

**Table 3:** A summary of treatment-related adverse events (AEs) with an incidence of ≥5% in the study population, and treatment-related select AEs

	All grade		Grade 3–4	
Treatment-related AEs, n (%)	n	Per cent	n	Per cent
Overall	64	84.2	17	22.4
Malaise	11	14.5	0	0
Pyrexia	11	14.5	0	0
Rash	11	14.5	0	0
Decreased appetite	11	14.5	1	1.3
Fatigue	9	11.8	1	1.3
Nausea	9	11.8	0	0
Pruritus	8	10.5	1	1.3
Hypothyroidism	7	9.2	0	0
Lymphocyte count decreased	7	9.2	3	3.9
Constipation	6	7.9	0	0
Diarrhoea	5	6.6	0	0
Dizziness	5	6.6	1	1.3
Arthralgia	4	5.3	0	0
Dermatitis acneiform	4	5.3	0	0
Hyponatraemia	4	5.3	2	2.6
Interstitial lung disease	4	5.3	2	2.6
Platelet count decreased	4	5.3	0	0
Rash maculopapular	4	5.3	0	0
Stomatitis	4	5.3	0	0
Vomiting	4	5.3	0	0
Treatment-related select AEs				
Endocrine disorders	11	14.5	0	0
Infusion reactions	3	3.9	0	0
Gastrointestinal toxicity	7	9.2	1	1.3
Hepatotoxicity	5	6.6	1	1.3
Pulmonary toxicity*	6	7.9	2	2.6
Nephrotoxicity	4	5.3	0	0
Skin toxicity	22	28.9	1	1.3

AEs and grade observed between the start date of the first administration of the study drug and 28 days after the last dose or the start date of subsequence anticancer therapy after the last dose whichever comes first were tabulated.

*Pulmonary toxicity included lung disorder.

Serious treatment-related AEs developed in 15 patients (19.7%); these events resolved or were resolving with appropriate treatment including steroid therapy or discontinuation of nivolumab (see online supplementary table S1).

Twelve patients (15.8%) were withdrawn from treatment due to treatment-related AEs (see online supplementary table S2). No treatment-related deaths were reported, but two patients (2.6%) died due to an aggravated underlying disease and cerebral infarction within 28 days after the last dose or shortly before starting post-study treatment after the last dose.

### Subgroup analysis

The subgroup analysis showed an association between ORR and smoking history. The ORR was significantly higher in current/former smokers than non-smokers (odds ratio [OR] 8.21; 95% CI 1.01 to 66.40). In contrast, other factors were not associated with ORR (table 4). The HR for PFS and OS for non-smokers relative to current/former smokers was 0.45 (95% CI 0.26 to 0.80; see online supplementary table S3A) and 0.73 (95% CI 0.40 to 1.35; see online supplementary table S3B).

**Table 4:** Subset analysis for IRC assessed ORR by baseline characteristics of the patients and PD-L1 expression level

Baseline characteristics/PD-L1 expression	Number of responders (n/N)	ORR (%)	95% CI	OR*	95% CI
Age (years)					
<65	12/40	30.0	18.1 to 45.4	2.66	0.83 to 8.49
≥65	5/36	13.9	6.1 to 28.7		
Gender					
Male	12/49	24.5	14.6 to 38.1	1.43	0.44 to 4.59
Female	5/27	18.5	8.2 to 36.7		
ECOG PS					
0	5/28	17.9	7.9 to 35.6	0.65	0.20 to 2.10
1	12/48	25.0	14.9 to 38.8		
Brain metastasis					
Yes	4/21	19.0	7.7 to 40.0	0.76	0.22 to 2.66
No	13/55	23.6	14.4 to 36.3		
Disease stage					
IV	13/62	21.0	12.7 to 32.6	0.66	0.18 to 2.46
Recurrent	4/14	28.6	11.7 to 54.6		
Smoking status†					
Yes	16/55	29.1	18.8 to 42.1	8.21	1.01 to 66.40
No	1/21	4.8	0.8 to 22.7		
EGFR mutation status					
Positive	1/20	5.0	0.9 to 23.6	0.13	0.02 to 1.07
Wild type or unknown	16/56	28.6	18.4 to 41.5		
PD-L1 expression level					
≥1%	9/27	33.3	18.6 to 52.2	1.67	0.37 to 7.60
<1%	3/13	23.1	8.2 to 50.3		
≥5%	9/19	47.4	27.3 to 68.3	5.40	1.18 to 24.64
<5%	3/21	14.3	5.0 to 34.6		
≥10%	9/18	50.0	29.0 to 71.0	6.33	1.37 to 29.20
<10%	3/22	13.6	4.7 to 33.3		
Not quantifiable	2/5	40.0	11.8 to 76.9		

*Odds ratio of first category relative to second category.

†Smoking status was classified as current/former smokers (yes) or never smokers (no).

ECOG, Eastern Cooperative Oncology Group; EGFR, epidermal growth factor receptor; IRC, independent radiology review committee; ORR, overall response rate; PD-L1, programmed cell death ligand-1; PS, performance status.

A total of 20 patients were positive for EGFR mutation. Although the ORR in these patients did not reach statistical significance (OR 0.13; 95% CI 0.02 to 1.07), it was higher in EGFR mutation wild type/unknown than positive patients (28.6% (n=16/56) vs 5.0% (n=1/20); table 4). Interestingly, one patient who had response to nivolumab in the EGFR mutation-positive group had a smoking history (see online supplementary table S4). The HR for OS of EGFR mutation-positive patients relative to EGFR mutation wild type/unknown patients was 2.10 (95% CI 1.14 to 3.88; see online supplementary table S3B). Further, stratification of ORR by smoking history and EGFR mutation status showed that EGFR mutation wild type or unknown/current or former smokers had highest response rate (ORR 31.9%, n=15/47), whereas none of EGFR mutation-positive/non-smokers responded to nivolumab treatment.

The association between response and OS was also examined. The survival rate at 24 months was 88.2% in the CR/PR (n=17) group, 36.8% in the SD (n=19) group and 13.5% in the progressive disease (PD, n=38) group (see online supplementary figure S2B). Median OS of patients with PD, SD, and CR/PR after nivolumab treatment was 12.4 months, 17.1 months, and not reached, respectively. PFS in patients with nivolumab treatment based on BOR is shown online supplementary figure S2A. Survivors with CR/PR at 24 months (n=15) included PD-L1-negative patients (n=2), EGFR mutation-positive patients (n=1), and non-smokers (n=1; see online supplementary table S4).

### PD-L1 subgroup analysis

Of the 76 patients included in the study, 45 (59.2%) patients had a tissue sample collected at baseline, of which the PD-L1 levels were quantifiable in 40 (88.9%) patients. The total number of patients positive for PD-L1 expression of 1%, 5%, and 10% were 27 (67.5%), 19 (47.5%), and 18 (45.0%), respectively. Higher PD-L1 status was associated with better outcomes (table 4, online supplementary table S5). The subset analysis for ORR, PFS and OS by EGFR and PD-L1 status is shown in online supplementary table S6.

## Discussion

In the present study, nivolumab treatment resulted in an ORR of 22.4%, with a median PFS of 2.8 months and median OS of 17.1 months. The median OS observed in this study was well above median OS values previously reported (12.2 and 9.9 months).^10^ ^12^ Tumour size shrinkage was observed in >50% of patients; patients with CR/PR showed long DOR and the treatment was well tolerated. These results clearly coincide with previously reported results of nivolumab and other immune checkpoint inhibitors.^10^ ^12–15^

Consistent with an earlier report,^12^ treatment-related AEs were reported in 84.2% of patients in this study. These AEs resolved after discontinuation of nivolumab or with appropriate treatment, with no treatment-related deaths. Thus, continuous monitoring of patients can ensure early detection and appropriate clinical management of any treatment-related AEs and maintain overall safety.

The Kaplan-Meier curve of responders showed that nearly 90% of patients survived at 24 months, suggesting that responders benefited with nivolumab treatment. Additionally, 36.8% of patients with SD survived at 24 months. Future studies with biomarker analysis are needed which can be expanded to both responders and patients with SD, if OS in these patients is significantly longer with nivolumab than with docetaxel.

The results of the study also showed that EGFR mutation status and smoking status were associated with greater efficacy of nivolumab. The ORR was significantly higher in current/former smokers than never smokers and positive EGFR mutations were associated with reduced OS in the treated patients. Thus, smoking and EGFR mutation status seem to be a predictive factor of nivolumab efficacy.

It has been reported that NSCLC in smokers has a distinct mutation signature leading to increased neoantigen generation which contributes to the efficacy of immune checkpoint inhibitors so that former or current smokers with NSCLC have a significantly higher response rate to PD-1 inhibitors compared with patients with minimal/no tobacco exposure.^16–18^ Assuming that NSCLC with a driver gene mutation may harbour fewer mutations than smokers, EGFR mutation-positive tumours may have less neoantigen and may be less responsive to nivolumab. However, in this study, characteristics of 17 patients who showed CR/PR with nivolumab suggested that EGFR mutation-positive patients or never smokers also respond to nivolumab. Thus, patient selection by smoking and/or EGFR mutation status needs to be carefully evaluated in the near future.

In the present study, although only 52.6% of patients were evaluated for PD-L1 status, the ORR of PD-L1-positive patients was higher compared with PD-L1-negative patients. These results are in accordance with previous studies, including one where nivolumab was associated with greater efficacy in PD-L1-positive patients while nivolumab and docetaxel had similar efficacies in PD-L1-negative patients.^10^ Despite some inconsistencies across studies, the association between higher clinical activity in response to PD-1/PD-L1 inhibitors and PD-L1 tumour overexpression was reported in a pooled analysis of seven clinical studies in pretreated NSCLC patients.^19^ In the present study, the rate of PD-L1-expression was higher compared with the Checkmate-057 study (67.5% vs 53.2%), which could be considered responsible for the longer OS. In contrast, the results of the present study showed that even PD-L1-negative patients responded to nivolumab treatment, suggesting that these patients may still benefit from nivolumab treatment.

The main limitations of the study were the small sample size and the absence of a comparator group; this study was designed as an open-label study with no control group with the intention of providing benefit to all patients included in the study.

Overall, the results of the present study are in line with the outcomes of the CheckMate-057 study and demonstrate that nivolumab is associated with clinical efficacy and manageable tolerability in Japanese patients with advanced/recurrent non-squamous NSCLC and patients with PD-L1-positive, current or former smokers, and patients with EGFR mutation wild type/unknown can experience greater benefit with nivolumab. Further studies with larger patient populations and comparator groups are warranted.
