# Supplementary material for: Multicentre phase II study of nivolumab in Japanese patients with advanced or recurrent non-squamous non-small cell lung cancer
Source: ESMO Open. 2017 Mar 7;1(4):e000108. doi: 10.1136/esmoopen-2016-000108 (PMC5566979; doi:10.1136/esmoopen-2016-000108)
Supplement: supplementary figures and tables [file esmoopen-2016-000108supp001.pdf]

Supplementary Figures

Figure S1. (A) Overall study design and (B) patient disposition during the study. AEs, adverse events; CR, complete response; PD, progressive disease; PR, partial response; SD, stable disease.

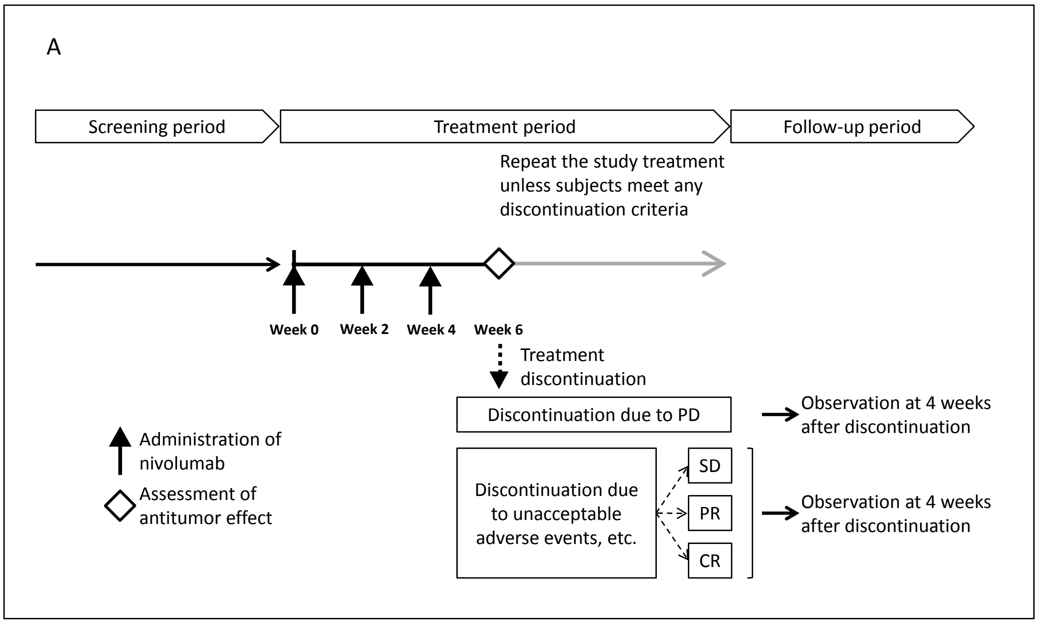

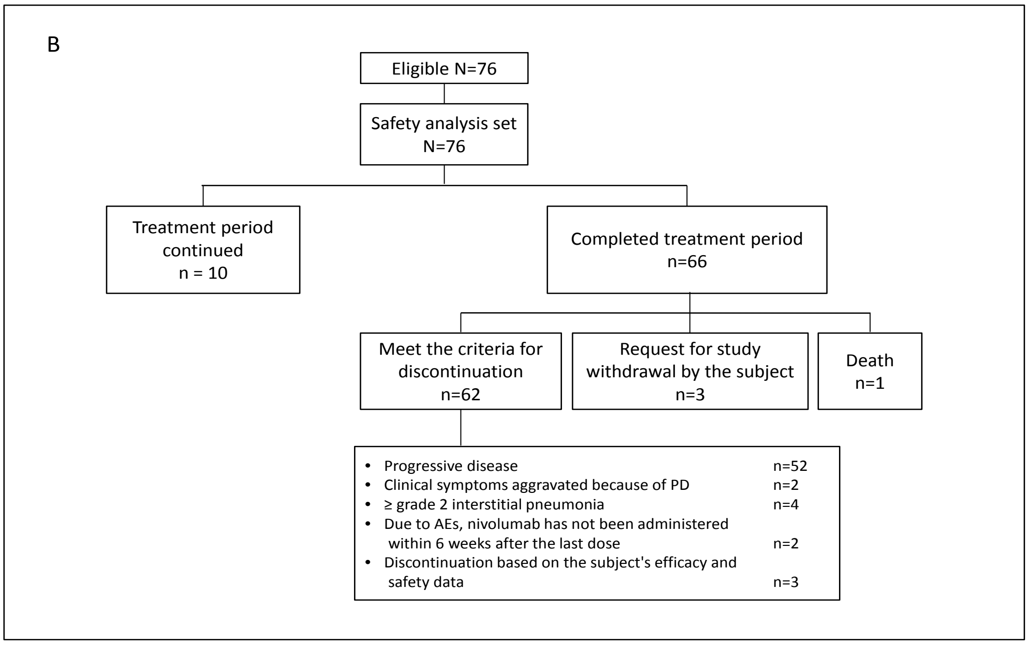

Figure S2. (A) Progression-free survival and (B) overall survival in patients with nivolumab treatment based on BOR. CR, complete response; PR, partial response; PD, progressive disease; SD, stable disease.

(A)

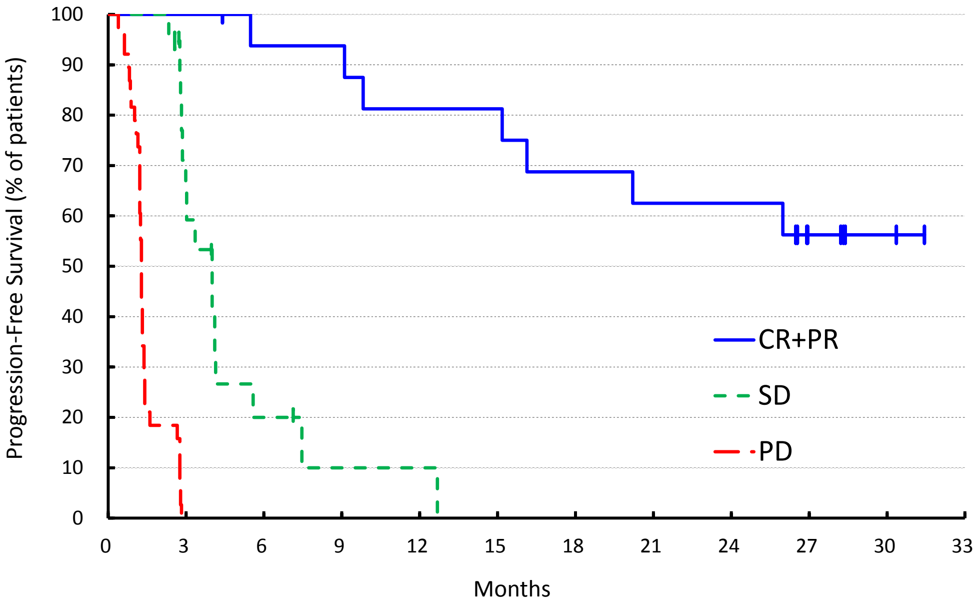

(B)

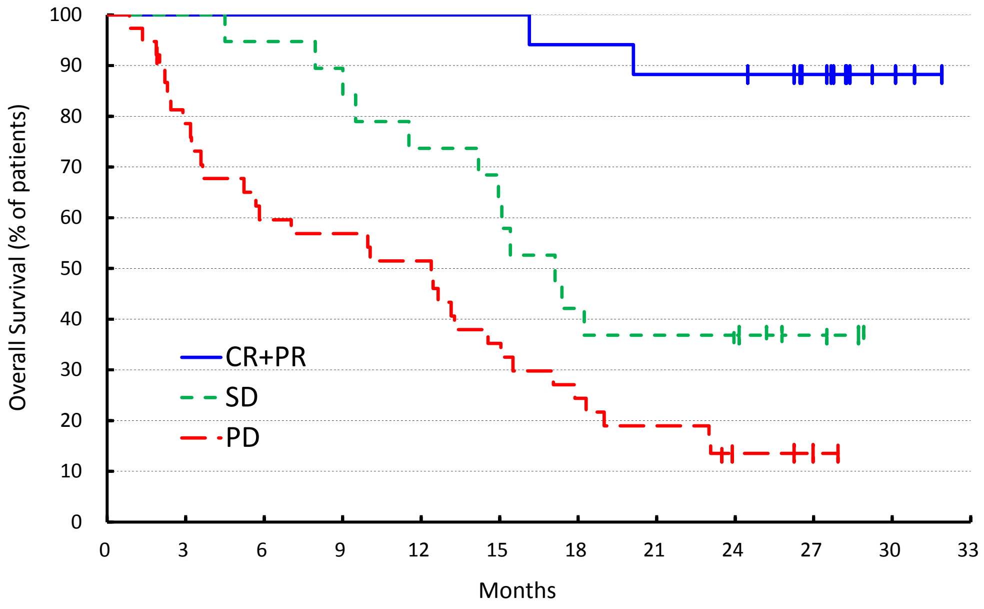

Supplementary Tables

Table S1. Serious treatment-related adverse events reported in patients treated with nivolumab.

**Serious treatment-related adverse events**	**All Grade**		**Grade 3–4**	
	**n**	**%**	**n**	**%**
**Overall**	15	19.7	10	13.2
Interstitial lung disease	3	3.9	2	2.6
Lung disorder	2	2.6	0	0
Pleural effusion	2	2.6	2	2.6
Secondary adrenocortical insufficiency	1	1.3	0	0
Colitis	1	1.3	1	1.3
Liver disorder	1	1.3	1	1.3
Bronchitis	1	1.3	1	1.3
Subdural hematoma	1	1.3	1	1.3
Decreased appetite	1	1.3	1	1.3
Cerebral infarction	1	1.3	0	0
Dizziness	1	1.3	1	1.3

AEs and Grade observed between the start date of the first administration of the study drug and 28 days after the last dose or the start date of subsequence anti-cancer therapy after the last dose whichever comes first were tabulated.

Table S2. Treatment-related adverse events leading to discontinuation of nivolumab.

Treatment-related adverse events	All Grade		Grade 3–4	
	**n**	**%**	**n**	**%**
**Overall**	12	15.8	6	7.9
Interstitial lung disease	4	5.3	2	2.6
Lung disorder	2	2.6	0	0
Pleural effusion	2	2.6	2	2.6
Thyroiditis chronic	1	1.3	0	0
Ascites	1	1.3	1	1.3
Colitis	1	1.3	1	1.3
Fatigue	1	1.3	1	1.3
Myalgia	1	1.3	1	1.3
Cerebral infarction	1	1.3	0	0

AEs and Grade observed between the start date of the first administration of the study drug and 28 days after the last dose or the start date of subsequence anti-cancer therapy after the last dose whichever comes first were tabulated.

Table S3. Subset analysis for (A) progression-free survival and (B) overall survival by baseline characteristics of patients.

(A)

Baseline characteristics	No. of events	n	Median (months)	95% CI	Hazard ratio[b]	95% CI
**Age**						
<65 years	28	40	3.4	2.7, 15.2	0.46	0.27, 0.77
≥65 years	33	36	1.4	1.2, 2.9		
**Gender**						
Male	35	49	2.8	1.4, 5.6	0.57	0.34, 0.95
Female	26	27	2.8	1.2, 3.0		
**ECOG PS**						
0	22	28	2.8	2.8, 4.1	0.80	0.48, 1.36
1	39	48	1.4	1.3, 3.0		
**Brain metastasis**						
Yes	17	21	2.8	1.3, 9.1	1.02	0.58, 1.79
No	44	55	2.8	1.4, 3.0		
**Disease stage**						
IV	54	62	2.7	1.3, 2.9	2.49	1.13, 5.49
recurrent	7	14	7.5	2.8, N.A.		
**Smoking status[a]**						
Yes	41	55	2.8	1.6, 9.1	0.45	0.26, 0.80
No	20	21	1.4	1.2, 2.8		
**EGFR mutation status**						
Positive	19	20	2.7	1.2, 2.9	1.89	1.08, 3.31
Wild type or unknown	42	56	2.8	1.4, 5.6		

CI, confidence interval; ECOG, Eastern Cooperative Oncology Group; EGFR, epidermal growth factor receptor; N.A., not applicable; PS, performance status.

[a]Smoking status was classified as current/former smokers (Yes) or never smokers (No).

[b]Hazard ratio of first category relative to second category.

(B)

Baseline characteristics	No. of events	n	Median (months)	95% CI	Hazard ratio[b]	95% CI
**Age**						
<65 years	21	40	25.0	15.0, N.A.	0.53	0.30, 0.95
≥65 years	27	36	14.6	10.1, 17.1		
**Gender**						
Male	30	49	17.4	12.6, N.A.	0.85	0.47, 1.53
Female	18	27	15.8	12.4, 23.1		
**ECOG PS**						
0	16	28	19.0	12.5, N.A.	0.75	0.41, 1.37
1	32	48	15.2	11.0, 20.1		
**Brain metastasis**						
Yes	16	21	15.5	5.8, 20.1	1.39	0.76, 2.54
No	32	55	17.1	13.1, N.A.		
**Disease stage**						
IV	43	62	15.4	12.4, 18.3	2.60	1.03, 6.58
recurrent	5	14	N.A.	12.6, N.A.		
**Smoking status[a]**						
Yes	33	55	17.4	12.6, N.A.	0.73	0.40, 1.35
No	15	21	14.8	10.1, 23.0		
**EGFR mutation status**						
Positive	16	20	14.2	5.7, 15.4	2.10	1.14, 3.88
Wild type or unknown	32	56	19.5	15.0, N.A.		

CI, confidence interval; ECOG, Eastern Cooperative Oncology Group; EGFR, epidermal growth factor receptor; N.A., not applicable; PS, performance status.

[a]Smoking status was classified as current/former smokers (Yes) or never smokers (No).

[b]Hazard ratio of first category relative to second category.

Table S4. Baseline characteristics of patients who responded to nivolumab treatment.

No.	BOR (IRC assessed)	Age (years)	Gender	ECOG PS	Smoking Status	EGFR mutation	PD-L1	OS (months)
1	PR	58	M	0	Former	Wild type or unknown	NA	27.5+
2	PR	64	M	1	Former	Wild type or unknown	≥10%	30.9+
3	PR	70	M	1	Former	Wild type or unknown	NA	20.1
4	PR	50	F	1	Current	Wild type or unknown	NE	30.1+
5	PR	72	F	0	Former	Wild type or unknown	<1%	16.1
6	PR	59	M	1	Former	Wild type or unknown	<1%	28.3+
7	PR	62	M	1	Former	Wild type or unknown	NA	27.7+
8	PR	64	M	1	Former	Wild type or unknown	≥10%	31.9+
9	PR	63	F	1	Former	Positive	NE	26.3+
10	PR	64	M	1	Former	Wild type or unknown	≥10%	28.4+
11	CR	67	M	0	Former	Wild type or unknown	≥10%	29.2+
12	PR	45	F	0	Non	Wild type or unknown	≥10%	24.5+
13	CR	57	M	1	Former	Wild type or unknown	<1%	26.5+
14	PR	59	M	1	Current	Wild type or unknown	≥10%	29.2+
15	PR	66	M	1	Former	Wild type or unknown	≥10%	27.8+
16	PR	64	M	0	Former	Wild type or unknown	≥10%	26.5+
17	PR	66	F	1	Former	Wild type or unknown	≥10%	28.2+

BOR, best overall response; ECOG, Eastern Cooperative Oncology Group; EGFR, epidermal growth factor receptor; F, female; M, male; NA, not available; NE, not evaluable; OS, overall survival; PD-L1, programmed cell death ligand 1; PS, performance status; +, a censored value.

Table S5. The progression-free survival (PFS) and overall survival (OS) by PD-L1 expression level in patients with nivolumab treatment.

	n	Months (95% CI)	Hazard ratio[a] (95% CI)
**PFS by PD-L1 level**			
≥1%	27	2.8 (1.3, 5.5)	0.84 (0.41, 1.71)
<1%	13	2.8 (1.4, 16.1)	
≥5%	19	4.4 (1.3, 26.5)	0.46 (0.23, 0.94)
<5%	21	2.7 (1.4, 2.8)	
≥10%	18	4.9 (1.4, 26.5)	0.41 (0.20, 0.85)
<10%	22	2.7 (1.3, 2.8)	
Not quantifiable	5	12.7 (1.3, 26.0)	
**OS by PD-L1 level**			
≥1%	27	27.0 (15.1, NA)	0.58 (0.25, 1.37)
<1%	13	14.6 (10.0, NA)	
≥5%	19	NA (13.1, NA)	0.49 (0.21, 1.18)
<5%	21	17.1 (12.5, 19.0)	
≥10%	18	NA (15.1, NA)	0.41 (0.17, 1.00)
<10%	22	16.6 (11.5, 19.0)	
Not quantifiable	5	NA (14.9, NA)	

CI, confidence interval; NA, not applicable; ORR, overall response rate; OS, overall survival; PD-L1, programmed cell death ligand-1; PFS, progression-free survival.

[a]Hazard ratio of first category relative to second category.

Table S6. Subset analysis for (A) overall response rate, (B) progression-free survival and (C) overall survival by EGFR and PD-L1 status.

(A)

EGFR	PD-L1 expression levels	No. of responders	n	ORR (%)	95%CI	Odds ratio[a]	95%CI
Positive	≥1%	0	9	0.0	0.0, 29.9	NC	NC
	<1%	0	4	0.0	0.0, 49.0		
	≥5%	0	4	0.0	0.0, 49.0	NC	NC
	<5%	0	9	0.0	0.0, 29.9		
	≥10%	0	3	0.0	0.0, 56.1	NC	NC
	<10%	0	10	0.0	0.0, 27.8		
Wild type or unknown	≥1%	9	18	50.0	29.0, 71.0	2.00	0.38, 10.58
	<1%	3	9	33.3	12.1, 64.6		
	≥5%	9	15	60.0	35.7, 80.2	4.50	0.85, 23.80
	<5%	3	12	25.0	8.9, 53.2		
	≥10%	9	15	60.0	35.7, 80.2	4.50	0.85, 23.80
	<10%	3	12	25.0	8.9, 53.2		

CI, confidence interval; EGFR, epidermal growth factor receptor; NC, not calculated; ORR, overall response rate; PD-L1, programmed cell death ligand 1.
[a]Odds ratio of first category relative to second category.

(B)

EGFR	PD-L1 expression levels	No. of Events	n	Median (months)	95%CI	Hazard ratio[a]	95% CI
Positive	≥1%	8	9	1.3	0.6, 2.8	1.27	0.37, 4.39
	<1%	4	4	2.7	1.3, 3.0		
	≥5%	4	4	1.1	0.6, 4.1	1.25	0.32, 4.95
	<5%	8	9	2.7	1.2, 2.8		
	≥10%	3	3	1.0	0.6, 4.1	0.87	0.18, 4.31
	<10%	9	10	2.0	1.2, 2.8		
Wild type or unknown	≥1%	11	18	5.5	1.4, N.A.	0.85	0.31, 2.29
	<1%	6	9	4.1	1.2, N.A.		
	≥5%	8	15	16.9	1.4, N.A.	0.49	0.19, 1.30
	<5%	9	12	2.8	1.2, 16.1		
	≥10%	8	15	16.9	1.4, N.A.	0.49	0.19, 1.30
	<10%	9	12	2.8	1.2, 16.1		

CI, confidence interval; EGFR, epidermal growth factor receptor; N.A., not applicable; PD-L1, programmed cell death ligand 1.
[a]Hazard ratio of first category relative to second category.

(C)

EGFR	PD-L1 expression levels	No. of events	n	Median (months)	95% CI	Hazard ratio[a]	95% CI
Positive	≥1%	8	9	13.1	2.2, 17.1	0.53	0.14, 2.00
	<1%	4	4	10.6	5.2, 14.6		
	≥5%	4	4	9.4	3.2, 15.1	1.93	0.54, 6.90
	<5%	8	9	14.2	2.2, 17.1		
	≥10%	3	3	13.1	5.7, 15.1	1.34	0.34, 5.21
	<10%	9	10	13.3	2.2, 17.1		
Wild type or unknown	≥1%	6	18	N.A.	19.0, N.A.	0.44	0.13, 1.48
	<1%	5	9	17.4	10.0, N.A.		
	≥5%	4	15	N.A.	15.5, N.A.	0.37	0.11, 1.29
	<5%	7	12	18.6	11.5, N.A.		
	≥10%	4	15	N.A.	15.5, N.A.	0.37	0.11, 1.29
	<10%	7	12	18.6	11.5, N.A.		

CI, confidence interval; EGFR, epidermal growth factor receptor; N.A., not applicable; PD-L1, programmed cell death ligand 1.

[a]Hazard ratio of first category relative to second category.

Table S7. Subsequent cancer therapy.

	n	%
Overall	41	53.9
Docetaxel	16	21.1
Tegafur, gimeracil, oteracil potassium	6	7.9
Pemetrexed sodium hydrate	3	3.9
Erlotinib	3	3.9
Docetaxel + bevacizumab	2	2.6
Paclitaxel	2	2.6
Vinorelbine	2	2.6
Tegafur, gimeracil, oteracil potassium + bevacizumab	2	2.6
Paclitaxel + carboplatin	1	1.3
Paclitaxel + bevacizumab	1	1.3
Gefitinib	1	1.3
Crizotinib	1	1.3
Selumetinib (AZD6244)	1	1.3
